# Exploring resident-staff relationships in nursing homes in Lebanon

**DOI:** 10.1080/17482631.2019.1688605

**Published:** 2019-11-12

**Authors:** Marina Gharibian Adra, Zepur Aharonian, Abla Mehio Sibai

**Affiliations:** a Rafic Hariri School of Nursing, Faculty of Nursing, American University of Beirut, Riad al Solh, Beirut, Lebanon; b Rafic Hariri School of Nursing, Faculty of Nursing, American University of Beirut, Beirut, Lebanon; c Department of Epidemiology & Population Health, Faculty of Health Sciences, American University of Beirut, Beirut, Lebanon

**Keywords:** Older residents, nursing homes, relationships, qualitative research, phenomenology

## Abstract

**Objectives**: To explore the prevailing relationships between residents and staff in nursing homes in Lebanon, and to elicit factors that influence these relationships.

**Method**: Using a qualitative phenomenological design, this study was conducted to explore the lived experience of residents, especially pertaining to their relationships with staff. The study included 13 residents aged 65 and above with no cognitive impairment. Data were collected using semi-structured interviews and were analysed using the Giorgi method.

**Findings**: Two main themes representing resident perceptions about their interactions with the nurses emerged: (1) relationships to satisfy the need for physical care, (2) relationships that foster a bond of caring and trust.

**Discussion**: Reflecting about resident-nurse relationships and examining factors that promote trust and stronger bonding help caregivers understand the importance of fostering a stronger relationship with residents. These findings have implications for developing policy and practice in nursing homes in Lebanon and elsewhere.

**Conclusion**: This is the first study conducted by a nurse researcher in Lebanon that has explicitly explored the nature of relationships between caregivers and care-receivers in nursing homes. The contribution of this study is not solely restricted to experiences and outcomes of care, but also includes implications for policy and practice.

## Introduction

While there is a consensus in the literature that positive relationships between older residents and those who care for them in nursing homes are built on mutual trust, understanding and sharing of collective knowledge, there is a dearth of studies that explain how these relationships develop and evolve over time (Brown-Wilson & Davies, 2009; van Stenis, Van Wingerden, & Kolkhuis Tanke, 2017). The transition from large extended families to small nuclear ones, accompanied by financial hardship and increased competing demands of raising children, has created a relative shortage in family members available for the provision of care for the older members. Hence, nursing homes are increasingly becoming an alternative formal means by which professional care is provided to the rapidly growing, ageing population. A nursing home is not only a health-care institution; it is also increasingly becoming a place where many residents live for the rest of their lives. Ensuring positive relationships in nursing home settings, therefore, is crucially important.

Lebanon, a high-income country, is the home for the largest proportion of older adults aged 65 years and over in the Arab region, constituting close to 9% of the population (Sibai, Rizk, & Kronfol, 2015). Waves of youth migration, coupled with the increased entry of women into the labour force and the conflicting demands of work and caregiving roles for younger generations, have impacted patterns of support for the old and led to the rise of formal, long-term care facilities in different forms and the increase in institutionalization rates of older adults into nursing homes. Moving to a nursing home involves a series of changes that can have adverse effects on older adults. Residents can become disconnected from symbols of their identity, social networks, familiar routines and meaningful belongings (Paddock, Brown Wilson, Walshe, & Todd, 2018). In addition, the transition to a nursing home often occurs at the nadir of physical and/or cognitive abilities (Kingston et al., 2017), thereby limiting residents’ functional abilities to adapt to this new context and increasing their reliance on care staff. Van Leeuwen et al. (2019) conducted a thematic synthesis of the perspective of more than 3400 older adults themselves and identified nine qualities of life domains, one of them being having close relationships which makes them feel supported and enable them to mean something for others. Ensuring a good quality of life in these institutions and promoting positive relationships with the staff in nursing homes can mitigate the adverse effects of institutionalization in older adults.

Only a limited number of studies have explored the relationship between older care recipients and caregivers in nursing homes in Lebanon. In 2012, a study conducted in two nursing homes in Lebanon explored the perceptions and perspectives of older residents about their quality of life and observed that the relationship between the residents and the nursing home staff was a key element in shaping the residents’ overall satisfaction with their new environment (Adra, 2013). Maintaining positive relationships with staff was most commonly identified by the older residents as bringing quality to their life. The nexus of their discussion on quality of life in the nursing home seemed to be their relationships with the staff members (Adra, 2013). These findings prompted us to pursue this premise further and to conduct a study exploring the prevailing relationships between residents and staff in nursing homes in Lebanon, and the factors influencing those relationships.

As elsewhere, there is a significant gap in our knowledge about the conditions of older people living in nursing homes in Lebanon, and about the challenge of providing a supportive environment that enhances positive relationships between residents and staff in these settings. Policy makers, practitioners and consumer groups need evidence and data on the factors that influence these relationships in such environments. Health service users have been identified as most appropriate to identify the strengths and reveal limitations of health service provision (Morris et al., 2017). Therefore, it was both a timely and necessary step to conduct this study with the aim of exploring the prevailing relationships between nursing home residents and staff, and the factors that influence these relationships from the standpoint of the older residents themselves, in order to design and shape supportive programs that aim at enhancing and personalizing service improvement. This research seeks to fill a significant gap in the literature and develop an interpretive framework to understand how relationships influence the experience of residents and staff in nursing homes.

## Background

Lebanon houses a total of 49 long-term care nursing homes that include around 4,180 residents, constituting less than 1.4% of the total number of older adults in Lebanon (Naja, 2012). While this proportion is considered one of the highest in the region, it remains very low when compared to percentages of older persons receiving formal care in institutions in Europe and the US (Sibai, Rizk, & Kronfol, 2014). The Lebanese population still has relatively strong family networks and cultural ideals continue to support intergenerational co-residence (Abdulrahim, Ajrouch, Jammal, & Antonucci, 2012). In several developed countries, older individuals often choose to reside in nursing homes (Sibai et al, 2015). In Lebanon, the social dimension of nursing home admissions exhibits cultural norms and values different than those seen in western countries. According to Lebanese cultural norms, older people generally have great expectations of help from their children, and one of their main fears is to end up living in a nursing home. The profile of nursing home residents is markedly different from that of Western countries (Sibai et al., 2015). Moving an older individual to a nursing home is considered in situations when the older individual has no one to care for him/her, or when she/he is seriously ill or demented that families can no longer assume responsibility for health care. Nevertheless, this will likely change as modern trends render working family members less available to care for their older family members.

Whilst several Western countries have instituted a system aiming for optimal resident care and adopted nursing home medicine as a unique speciality, the situation is not the same in Lebanon. The number of geriatricians in the country does not exceed 15, yielding one geriatrician for every 20,000older persons over 65 (Tohme & Hajjar, 2015), the nature of medical and nursing training is not directed towards a holistic model of patient-centred care, making the need for interdisciplinary team-based approach crucial in Lebanon. Accreditation guidelines for the nursing homes in Lebanon are currently being developed by the ministry of Social Affairs and the standards being examined for alignment with those issued by the Ministry of public Health to ensure consistency (Sibai, Rizk, Costanian, & Beard, 2016; Sibai et al., 2015).

Care in nursing homes has different components that strongly influence the quality of life for residents, including proficiency in caregiving practices, autonomy, individualized care, communication and relationships with caregivers and staff (Adra, Hopton, & Keady, 2015; Custers, Westerhof, Kuin, Gerritsen, & Riksen-Walraven, 2012). Bergland and Kirkevold (2005) and Bergland and Kirkevold (2007) discussed how the relationship between caregivers and residents is asymmetric, with nurses and caregivers being in a position of control over residents (Brown-Wilson & Davies, 2009; Cohen-Mansfield & Parpura-Gill, 2008). On the other hand, some studies have shown symmetric relationships between staff and residents, where the two sides are mutually dependent in their interactions (Dewar & Nolan, 2013). To date, several studies have shown that the personnel in the nursing home are among the elements conditioning the life of the resident (Squires et al., 2015) influencing their social circle (Brown-Wilson, Swarbrick, Pilling, & Keady, 2013), mental attitude, adaptability and sense of security (Chuang, Abbey, Yeh, Tseng, & Liu, 2015). For the older residents, reciprocity proved the existence of good relationships, and staff continuity facilitated the establishment of good relationships between the older residents and their care providers since it paved the way for the development of reciprocal relationships (Adra, 2013).

In general, encounters between residents and staff can have either positive or negative effects (Roberts & Bowers, 2015; Westin & Danielson, 2007). Studies from Finland and Sweden report that, for the resident, life’s meaning is strongly influenced by interpersonal relationships (Lung & Liu, 2016; Takkinen & Ruoppila, 2001; Westin & Danielson, 2007). Other studies from Lebanon also note that, for some residents, establishing a close relationship or friendship with staff is of great importance (Adra, 2013; Adra et al., 2015). The findings of the study reported by Kehyayan, Hirdes, Tyas, and Stolee (2015) confirm that resident-staff bonding remains a substantial area of concern for the majority of residents. Taken together, the available data highlight the importance of relationships and encounters between residents and staff in how residents experience care in nursing homes (Custers et al., 2012). This is especially pertinent to Lebanese culture, in which social interactions are a particularly important aspect of people’s lives (Adra et al., 2015).

While the literature addressing relationships between nursing home older residents and staff exists in Western societies (Brown-Wilson, 2008; Brown-Wilson & Davies, 2009; Crespo, Bernaldo de Quiros, Gomez, & Hornillos, 2012; Hauge & Heggen, 2007; Kehyayan et al., 2015; Palacios‐Ceña et al., 2013), little is known about the subject in the Arab region in general and in Lebanon in particular.

The purpose of this paper is to explore the quality and meaning of such relationships in the nursing home setting. More specifically, we examine the behaviours and personal perspectives of the residents within the context of nursing homes to gain an in-depth understanding of how relationships with staff are developed and experienced.

## Methods

### Design

This study was conducted using a qualitative phenomenological design to explore the lived experiences of nursing home residents, especially pertaining to their relationship with the staff. Qualitative studies are typically used to achieve a deeper understanding of and find explanations for people’s behaviours under specific circumstances (Horsburgh, 2003; Kuper, Reeves, & Levinson, 2008). The main characteristic of this method is that the researcher is intimately involved in data collection and analysis (Denzin & Lincoln, 2005). Data collection requires the researcher to interact with the study participants and with their social context (Piot & Garnett, 2009). In the field of qualitative studies, phenomenology attempts to understand how individuals construct their world view (Denzin & Lincoln, 2005). The aim of phenomenology is to identify the essence of the experience lived by participants (Giorgi, 2005; Giorgi & Giorgi, 2003), which is the subjective reflection of human beings when taking part in events in a specific geographical, social and cultural environment (Reeves, Albert, Kuper, & Hodges, 2008). This approach allowed the participants, through in-depth interviews, to elicit their own meaning of their experience (Polit & Beck, 2014), and provided the opportunity for older residents to share their lived experiences with the researcher, without imposing the views of the researcher. The purpose was to explicate the structure or essence of the lived experience in the search for meaning that identifies the essence of the phenomena, and its accurate description through every day’s lived experience. (Giorgi, 2005; Streubert & Carpenter, 2011).

### Sampling strategies

The first phase involved purposeful sampling which is considered by Patton (1990) as the most important kind of non-probability sampling, to identify the primary participants. To gather information from the residents themselves (Denzin & Lincoln, 2005), I selected the sample based on my judgement and the purpose of the research looking for those who have had experiences relating to the phenomenon to be researched after several observation visits to the sites. According to Patton (1990), the “logic and power of purposeful sampling lies in selecting information-rich cases for study in depth. Information-rich cases are those from which one can learn a great deal about issues of central importance to the purpose of the research” (p. 169). The second phase involved in-depth interviewing of the remaining residents to gain a deeper understanding of certain issues and trends detected during the first phase (Kuper et al., 2008). The sample was recruited by the researcher from two nursing homes in Beirut. The decision of which participants would be approached to take part in the study was made in consultation with the geriatrician in charge, the family caregiver, the nurse manager, and the researcher’s own assessment.

The inclusion criteria for older residents comprised: 1) being aged 65 years and over; 2) having been a resident in a nursing home for more than 6 months; 3) having the ability to communicate verbally; and finally, 4) being able and willing to consent to and engage in the research study. As such, mentally or physically impaired individuals with debilitating conditions were excluded from the pool of potential participants. These included older residents with dementia who were severely agitated or very distressed and were unable to give informed consent, residents with a terminal health condition, such as cancer, or severe disabling stroke who are unlikely to recover, and for whom intensive palliative care is the predominant focus of goal of care for the time remaining. An older resident was also considered ineligible to participate if he/she could not understand information, retain information, or communicate his/her consent.

Sample size was determined by theoretical saturation of the data. Phenomenological studies often rely on very small samples—typically between eight and 15 participants (Polit & Beck, 2014).

## Data collection

Following approval of the study by the Institutional Review Board (IRB) of the American University of Beirut and the administration of the two nursing homes, recruitment started.

The two nursing homes were selected because they run relatively comprehensive services including rehabilitative, preventive, and curative services. Both nursing homes house older people with and without dementia and were chosen on the basis of their geographic accessibility, size, and willingness of their administrations to take part in the research study. The geriatrician and the nurse manager were approached for identifying eligible participants based on the criteria stated above. According to Bentz and Shapiro (1998) “Doing phenomenology” means capturing “rich descriptions of phenomena and their setting” (p.104). For this reason, the actual research questions that were put to participants were: (a) how would you describe your relationship with the nursing home staff? Tell me about your experience. (b) Is it important for you to maintain relationships with the nursing home staff? Why? A follow-up probe was often helpful. My questions were directed to the participant’s experiences, feelings, beliefs and convictions. I focused on “what goes on within” the participants and got the participants to describe the lived experience. This approach allowed the researcher to understand the world from the subjects’ point of view (Murray et al., 2009), to unfold meaning of phenomena in their own terms-to provide a description of human experience as it is experienced by the person himself. The aim was to look for emerging themes and topics that could be further expanded on during the second phase of the study.

Data of the first phase were analysed and themes abstracted, then the second phase probed deeper into the findings of the first phase to arrive at a deeper understanding of the relationships experienced and factors affecting them. The researcher used “bracketing,” which in phenomenology research indicates retaining beliefs and involves using the phenomenological reduction method; this approach allowed a critical examination of the phenomena without the influence of the researcher’s own beliefs (Dowling, 2007).

The semi-structured interviews were based on an interview guide, which was revised after reviewing the residents ‘accounts obtained during the purposeful sampling, aimed at eliciting further information regarding specific themes and topics of interest that had emerged from the first round of interviews (Kendall et al., 2009). New participants were interviewed until data saturation was achieved, that is, until the quality, completeness, and amount of the information was sufficient, the topic was exhausted or saturated, and no new themes were elicited in the interviews. A total of 13 participants were interviewed, six men and seven women, with age ranging from 65 to 78 years. Field notes were also taken during the interviews. All the interviews were tape-recorded and transcribed verbatim. The first two interviews were conducted by the primary investigator in the presence of a research assistant who then continued conducting the other interviews. All the interviews were conducted in Arabic. The interviewer used communication skills such as reflection, nodding, questioning, clarification, and maintaining eye contact to facilitate and encourage participants to talk, until there were no new themes or issues emerging from the participants.

## Data analysis

The audio-taped interviews were transcribed verbatim and translated into English. An independent bilingual person not familiar with the study back translated the interviews into Arabic. The original and back-translated versions were compared for semantic equivalence. Data collection and analysis were done simultaneously. The Giorgi method was used to implement qualitative data analysis (Giorgi, 1997, 2009). Each interview was read completely at first and then line by line. Holistic reading allowed the identification of the main statements on the participants’ experience. This provided the researcher with an opportunity to interact with details embedded in the data and thereby to grasp the underlying meaning of these data. Next, the researcher went back to the beginning and read through the interviews once more based upon a process of meaning discrimination, constituting “meaning units” from within the perspective and with focus on the phenomenon being researched. The end of this step was a series of meaning units still expressed in the participants’ own everyday language. Once the meaning units were established, transformation of the participants’ everyday language was required. (Giorgi, 1997). In this step, the statements of the participants were transformed by the principal investigator (PI) to express the insight contained in them more directly. Finally, the PI synthesized all of the transformed meaning units into a consistent statement regarding the participants’ experience. To guarantee an accurate comprehension of participants lived experience and to maximize bracketing of bias (Seidman, 2006), the participants were visited a second time to present both the textural and structural description. This process of member-checking is considered crucial for establishing credibility (Lincoln & Guba, 1985). All participants agreed to member-checking. In this study, the PI stayed as close as possible to the language that the residents used in their accounts conveying the residents’ experiences of their life in the nursing home. The PI incorporated the residents’ own perceptions and interpretations by applying terms or perspectives that best captured them. The method was discovery-oriented rather than verification-oriented.

To evaluate dependability of the study data, the PI analysed all interviews. A second investigator with experience in qualitative research analysed 20% of the data, and the results were compared. Triangulation was attempted by linking the observational data with the interview data: convergence of the two sources of data suggested validity of the data.

## Human subject consideration

Older residents were informed of the voluntary nature of participation so that they could withdraw from the study at any time without having the care delivered to them affected. Those who agreed to participate were asked to sign a consent form and were assured of strict confidentiality. Data were anonymized via the adoption of a code to identify each participant, and pseudonyms were used for qualitative quotes. It was made clear that the interviewer or the participant could discontinue the interview whenever they wished or when the interviewer deemed it necessary due to their discomfort, fatigue, or distress. Participants were also given the freedom to stop the tape at any time. All interviews were conducted in a private room in the nursing home, and the interviewer made sure that the staff were not around while the interviews were conducted, in order to avoid social desirability bias and any sensitivity of the participants, so that they were able to share their experiences freely without any discomfort.

## Findings

From our analysis, two main themes representing resident perceptions about their interactions with the nurses at the nursing home emerged: (1) interaction with the nurses to satisfy the need for care of the physical type; and (2) interaction with a bond of caring and trust.

## Theme I: interaction with the nurses to satisfy the need for care of the physical type

1. Basic Functional Care Needs

From the older residents’ perspective, their main functional care needs comprised personal hygiene, nutrition and exercise. As the name of this property suggests, it has to do with the fulfilment of their main physical functions. The inability to control parts of the body indicated the need, to varying degrees, for nursing care of the physical type. The changes in bodily functioning called for nursing care that the older persons expected to be given in the form of support and encouragement. The personal hygiene of the nursing home residents was maintained through the performance of the daily tasks of bathing and grooming, which often involved intimacy, i.e., touching, handling or examining genitals, offering assistance with incontinence pads, going to the toilet, washing, dressing and undressing. Care was delivered to them whenever needed, nurses responded to their calls, the treatment they got was humane, they felt they were in safe hands, and their experience of the nursing care was positive:

“I was going out of the bathroom when I fell down and broke my hip. I also have arthritis, so I am unable to walk without my walker. I thank God I am surrounded with these nurses who help me in everything: walking, changing my clothes, bathing etc.”

(Interview: R 8)

“You know I have been bedridden for too long. They bathe me, they feed me, they take very good care of me and I accept all this because there is no other solution. There is nobody at home who can take care of me.”

(Interview: R10)

Answering a question about his relationships with the staff, one resident said:

(Interview: R7)

“I cannot get out of this chair. My legs are paralyzed. Sometimes I don’t even want to go to the bathroom. But thank God I have social ties with those boys and they help me satisfy my physical needs. I am very thankful to them.”

The question of food appeared in participant’s list of priorities, and coffee breaks and mealtimes were the “bright parts of the days” in the everyday routine of the nursing home, hence their focus and lengthy comments on food.

“Food is very good. They prepare all kinds of food and it’s so tasty. Yesterday we ate fish. I eat everything. Thank God I don’t have diabetes or hypertension. I can eat all kinds of food. I have no restrictions.”

(Interview: R5)

Coffee breaks and mealtimes presented great opportunity for chatting and exchanging information and paved the way for the development of social relationships. These activities proved to be particularly enjoyable to the residents. They created the opportunity for socialization. They could build friendships with staff as well as with other residents which satisfied their need for social relatedness. Some residents demonstrated through social exchange with staff that they wished to develop personal relationships.

Taking a walk was the kind of exercise often performed by the residents with the ability to move. The safety and length of the corridors and the hallways outside the nursing home encouraged the residents to go on a walk. Some reported doing walking exercises and stressed their importance, even those who were wheelchair users. The friendly attitudes of the nurses and the fact that they were dependable mattered a great deal, which made participants feel certain that they would be by their side in case of illness or pain.

*“As soon as I have my coffee at 3:00 p.m., I go outside to breathe fresh air. I walk (sitting in wheelchair) there and I smoke a cigarette*.”

(Interview: R4)

“Maintaining optimal levels of physical activity might minimize the loss of my physical capabilities and retain my functional capacity.”

(Interview: R7)

Patience, the exhibition of attention and love, and keeping a good temperament and pleasant behaviour during the delivery of care and while satisfying residents’ basic functional care needs represented the older residents’ expectations from staff members and provided them with considerable support.

Communication with the nursing staff and the monitoring of their verbal and nonverbal expression allowed the older residents to make a judgement about the personal attributes of their care providers. Some of them stated that being treated with consideration made them feel warm and valued:

“They take very good care of us. May God bless all the nurses and the doctors, and may the Almighty keep them safe. They keep checking on us as you can see. I am happy. Currently it’s better than home.”

(Interview: R10)

But one resident reported the negative experiences she had in the nursing home by emphasizing the importance of the nursing staff displaying a caring attitude:

“They (the nurses) are very good at providing physical care, but they do not cater well to emotional side of things … ”

(Interview: R1)

The residents expected to receive care, kind-heartedness, warmth and tenderness from their caregivers. Some of the residents wanted to have their personal biographies recognized and valued. They shared their stories with nurses during care routines in an attempt to develop more personal relationships with staff.

(2) Safety Needs

The older residents experienced some level of anxiety and fear about dealing with unpredictable illnesses or sudden incidents. If help was not provided immediately when they asked for it, they were dominated by a feeling of insecurity. As a result, the ready accessibility to nursing staff, nursing care, and medical resources improved the residents’ feelings of security. Older residents mentioned that the nurses’ visits and having the opportunity to chat with them made them feel secure and helped them to alleviate any discomfort:

“I haven’t seen the nurse yet today. She comes and visits me every day. When I see her today I will tell her about my pain. I am sure she will give me some medication to take the pain away.”

(Interview: R2)

“It’s safer for me to live in this nursing home. At home I have no body to take care of me. I don’t think it’s safe for somebody of my age to live alone at home.”

## Theme II: interaction with a bond of caring and trust

This type of relationship was established by:

*Staff respecting the older person, sustaining their identity, individualized care and continuity of staff*

1. Respecting the older person: the culture of Lebanon emphasizes the concept of respecting older people and they, in turn, expect to be respectfully treated. In Lebanese society, older people are looked upon as a source of spiritual blessing and models of religious faith, wisdom, and love. Respecting represented the staff members’ general method of interaction.

For residents, the technical details of care, such as passing food trays, making beds, assisting with bathing and personal care, occupied a secondary position in comparison with the indicators of personalized affection and friendship they perceived in the care given to them. One of the residents reported that certain staff members delivered care with an uncaring spirit, which created a feeling of being a nuisance on the part of the older resident whenever she wanted to ask for help:

“She is very good at providing physical care but she does not care about the emotional side of things … She just gives me my medications and disappears.”

(Interview: R8)

In their assessment of the quality of care, they referred to their relationships with the nurses and underlined the degree of closeness to their caregivers.

“My relationship with the staff? It is very good. The director here is so sweet. God bless her! She makes sure that everyone in the institution is satisfied. I am so thankful to her, so thankful. I always pray for her, I love her so much.”

“Most staff are keenly aware of the details of care that are significant for individual residents, and they work hard to ensure that care is delivered according to our wishes.”

(Interview: R9)

Residents described their confidence that most members of staff would do as they said they would:

“I always say I feel safe with her. If I ask her for something, she does it. Some of them they take ages. She’ll say I’ll go and get somebody and you know that she will come back with somebody very soon, she’s very good.”

(Interview: R13)

It was important for the older residents that staff respect them and provide care to them by fostering positive attitudes towards ageing and older residents. They were concerned about the perception that working with older residents lacks value and status in society. The description participants gave of “good care” referred to the provider as someone who “really liked his/her work” and “really cared about the older residents.” They underlined the need to promote the concept of working with older residents positively.

“Nurses should love older people in order to work with them; they should spend time with them. They should not feel they are forced to come and take care of them. Imagine that we gave birth to these younger generations, helped them to start their lives and now they are helping us to finish ours. We helped them face the world and succeeded; and now they are helping us to die in return. Do you see the difference?”

(Interview: R3)

The residents also cared about intimate relations with staff, which meant staff holding them, kissing them, making them feel that they are loved. According to the older residents, empathy was considered to be the central component of a caring interaction. Receptive, reliable, and continuous communication with the staff was regarded as a significant indicator of a well-functioning caregiving interaction. This included conveying correct information, showing sensitivity to the older resident’s views and needs, and giving them kind treatment. Residents thought that empathy involving an understanding of their situation, perspective, and feelings was a necessary basis for constructive and trusting relationships with staff. Such qualities appear to be important prerequisites for establishing a sense of security among the older persons and for building trusting relationships between them and the staff members. According to their descriptions, they strove for reciprocity and empathic understanding as these extracts convey:

“I am becoming very attached to them and I love them, just love them as my own grandchildren. For me, to have someone next to me, to have someone’s attention, that is the most important thing that I want to make sure I have.”

(Interview: R4)

“They take good care of us. May God bless the director, the nurses and the doctors. May the Almighty keep them safe. They always watch over us and keep checking on us. As you can see, we are like a family and I am very happy in this nursing home.”

(Interview: R11)

They seemed to prefer a close friendship over transient or superficial encounters and a relationship bond was essential to full satisfaction. Bonds marked by positive concern and caring offered satisfaction to older residents. A bond of caring and trust supported the most positive experiences for residents.

(2) Sustaining the identity of the older person: using previous knowledge about the older resident and through shared experiences and narrated parts of the resident’s life story, the nurses mostly succeeded in sustaining the older resident’s identity. Participants commented that this approach helped to develop a common bond between themselves and staff:

“She doesn’t see me anymore as a patient, she sees the person behind that. I tell her about myself and she tells me about herself and it connects with things in your life as well.”

(Interview: R12)

Care staff spent time getting to know the residents and were willing to meet their individual needs. In addition to showing concern to the older resident, they used alternate forms of interaction such as nonverbal active listening, therapeutic touch and observing individual responses of the older resident. These techniques facilitated development of a trusting relationship and demonstrated an empathic understanding of the older resident which directed the staff member to act upon their concern. It was very important that caring, concern, and affection be mutual and reciprocal for bonding to develop.

“Yes, I am so attached to some of them. For example, when they go on vacation I miss them and ask about them. I feel as if they are my children; they are so important in our lives that they ask us about our families and kids. They care about us, and if one of them is transferred to another floor we ask about him/her because we miss that person.”

(Interview: R13)

“You need people who are compassionate, that is the most important thing. Yesterday he shaved me, and he did a very good job. I enjoyed it because we had never bonded before and I felt we bonded.”

(Interview: R4)

“They give me plenty of love, and I thank God for sending me to this place because it is really what I needed. They add so much to my life and keep me from being lonely.”

(Interview: R11)

Older residents seemed to appreciate the emotional capacity of the staff to care about residents.

They used key expressions such as affection, motivation, and genuine friendship to describe their experience in these relationships:

(3) Individualizing care

Older residents considered individualized care central for facilitating meaningful communication and interaction with staff. The primary goal in individualizing care was to have as few rules as possible. Many residents identified the need to “know the person” as an important prerequisite to individualized care. “Knowing the person” reflected the process whereby nurses came to perceive a resident as someone more than an old person, to respond to their individual needs, and to try and bring a personal dimension to care. Older residents wanted to have their personal biographies recognized and valued as a basis for individualized care. They mentioned that they enjoyed talking about their lives, and that being listened to accorded “personhood and significance” to them. An older resident described an example of care that involved “doing something” for a resident that is important to them individually. An example illustrates the case of an older resident who enjoys listening to classical music and is given the opportunity and the means to listen to it:

“I love listening to classical music. The nurses have arranged for me to buy a tape recorder so I will be able to listen to classical music.”

(Interview: R2)

This approach resulted in care that accorded with the resident’s personal meanings and values and enhanced staff-resident relationship.

The older residents stated that staff members were generally kind and helpful. They emphasized how hard they worked and that “nothing was too much trouble” for them. Knowing the nurses by name formed the basis for a relationship and for trusting the care. For the residents, reciprocity, mutuality and trust were crucial features of good, beneficial, and satisfying relationships.

Moreover, showing care and loving-kindness, having warmth and affection, and feeling and exhibiting concern described the expectations the older residents had of the nursing staff. Some of the participants stated that someone showing consideration for them made them feel warm and valued, and this contributed to the development of bonding and a trusting relationship between them.

“I know that I am comfortable in here and that the employees are so sweet. They love me and they treat me as if I were their mother or their grandmother. They are very gentle when they’re handling me. Well, I am thankful to all.”

(Interview: R10)

“I am becoming very attached to them and I love them, just love them as my own grandchildren. For me, to have someone next to me, to have a confidant, to have someone’s attention that is the most important thing that I want to make sure I have.”

(Interview: R7)

The positive attitudes of the staff, including showing respect and caring for the residents, provided the older residents with considerable emotional support. They liked to be listened to, valued, respected and loved and to experience continuous interaction with staff. These activities become a foundation for the development of personal relationships.

(4) Time and continuity of staff

Time was considered essential to forging trust, a key component of relationships and care provision, as one of the residents explained:

“If she is giving me a bath and she is rushing, I wouldn’t want to take a bath. If she has the time to sit and talk with me it makes me feel that I can trust her. That is important to have someone who you can trust.”

(Interview: R9)

Lack of time the staff members had to converse with residents and lack of continuity of care represented a problem in forming meaningful relationships with staff. One of the residents complained about the transfer of staff she knew to other units. When an inquiry was made about the reason behind the transfer of staff members from one unit to the other, it was mentioned that the reason was because the other unit was “understaffed.” This disruption constituted a hindrance to the development of relationships with nurses. An older resident described a special relationship she had with one of the staff members:

“There is this pretty young girl … she comes in when she is on duty, washes me, she puts me to bed and then she asks me if I want anything else. I like her as I like my grandchildren and she treats me not just like a patient but she sees me like her grandma. She knows intimate details about me and she knows my likes and dislikes. But now they transferred her to another unit. I truly miss her.”

(Interview: R7)

Staff continuity facilitated the establishment of relationships since it presented greater opportunity for chatting and exchanging information and paved the way for the development of reciprocal relationships.

“I want the same nurse to take care of me consistently because you really have good relationships with her/him. I feel more comfortable and I like to know who to expect.”

Older residents enjoyed showing their picture albums to staff and telling them about themselves and their family. Stories shared by residents with staff made a major contribution towards the development of personal relationships. Such stories helped the staff to know the resident better and understand their biography, and this supported them in creating personalized care routines appropriate to each resident.

## Discussion

This research sheds light on the nature of relationships between nurses and the residents they care for in nursing homes in Lebanon and the contextual influences that shape these relationships. Our results show that residents and staff experience continuous interaction with each other throughout the day. While many of these interactions derive from fulfilment of the main physical functions of the older residents, the findings point to situations where residents actively seek opportunities to interact with staff in a more personal manner. These results agree with previous studies (Ball, Lepore, Perkins, Hollingsworth, & Sweatman, 2009; Dewing, 2004; Palacios- Ceña et al., 2013).

In our results, we observed that it is the nurses who initiated socialization and defined the nature of conversations. In this regard, our results coincide with those from previous studies (Bergland and Kikevold, 2005; Casey, Low, Jeon, & Brodaty, 2015; Wadensten, 2005). When nurses favoured topics of conversation that are centred on issues of care and on the illnesses affecting the residents, then unsentimental relationships developed. Similarly, if care focused on the physical needs of the residents, rather than what was important to the older person, there was evidence to suggest that it provided the least positive experiences for the residents within this study.

Uncompassionate relationships between residents and staff most commonly developed during the care routines within the nursing home, when communication revolved primarily around caregiving tasks. It was generally the focus on what needed to be done rather than the significance of the task to that person that resulted in this situation.

All residents in the present study wanted good care, which was described in terms of the residents’ needs support with personal hygiene, eating, drinking, ambulating and exercising. This has been found in other studies (Drageset, Haugan, & Tranvåg, 2017; Rantz & Zwygart-Stauffacher, 2004; Sacco‐Peterson & Borell, 2004; Stabell, Eide, Solheim, Solberg, & Rustoen, 2004) describing a quality institution as one where visible care such as ambulation and toileting is seen to be happening. Bowers, Fibich, and Jacobson (2001) also describe how some residents focus on the technical aspects of care and judge the quality of the care in terms of their own expectations of how care should be delivered; as noted in other studies as well (Cho et al., 2017; Rodríguez-Martín, B., Martínez-Andrés, Cervera-Monteagudo, Notario-Pacheco, & Martínez-Vizcaíno, 2013; Shippee, Henning-Smith, Kane, & Lewis, 2013; Taylor, Sims, & Haines, 2014). However, many residents used the care routines to provide information about themselves to staff. They shared personal stories with staff using the task that was being undertaken as a starting point. These stories suggested that some residents were keen to develop more personal relationships with staff.

From the perspectives of residents, the factors that positively influenced the development of bonding, caring and trusting relationships included: being respected by the nurses, continuity of staff, maintenance of their identity, and receiving individualized care, congruent with the findings of McGilton and Boscart (2007) and James, Blomberg, and Kihlgren (2014) who concluded that the factors that positively influenced the development of close relationships included the care provider’s technical competence, interaction and communication, closeness in the relationship, caring and engagement, fulfiling the needs of the resident, knowing the resident, and care provider’s being reliable and trustworthy.

When staff adopted a caring attitude towards the resident with a bond of caring and trust, residents were very active participants in relationship development. Moreover, there was some evidence to suggest that these methods supported the most positive experiences for residents. Bonding was also enhanced by continuity of staff which implied feelings of attachment and affection. Many residents valued the development of an interpersonal bond or relationships marked by stability and affective concern which were developed when staff demonstrated biographical knowledge about the resident (meaning that staff understood the type of person the resident had been and what was important to them now.) The process of finding out what mattered to each resident, having caring conversations with the resident moved the focus beyond the instrumental notion of physical care to understanding the implications of care for that person; contrary to the results reported by Iwasiw, Goldenberg, and Nancy (2003), who found that relationships between residents and staff seemed formal and existed out of necessity. Dewar and Nolan (2013) generated a model called the 7 “C”s that captures in detail the factors necessary to promote “appreciative caring conversations” that are the route to compassionate care. Such conversations represent an advanced and highly skilful form of relational practice that is often not fully recognized within the culture that currently dominates nursing homes in Lebanon.

Relationships have been found to be enhanced for residents if caregivers are reliable, empathic, and consistent in their approach (Dewar & MacBride, 2017; McGilton et al., 2003; Sandberg, Nolan, & Lundh, 2002; Smebye & Kirkevold, 2013), similar to the findings in this study where the resident had to believe that the caregiver cares about his/her welfare and likes him or her. Having some intimate bond appeared to be important and necessary for the happiness of resident. Moreover, they felt at home when such relationships were built between them and staff. Canham et al. (2017) report similar findings in their study, which emphasized the importance of interpersonal relationships in creating a homelike environment within institutional settings.

Previous research has also demonstrated the importance of personal relationships between residents and staff and has suggested that the development of relationships often occurs within the context of care provision (Brown-Wilson, 2008; Brown Wilson, Davies, & Nolan, 2009). Brown-Wilson (2008) provided a typology of potential relationships in care home settings: (a) pragmatic relationships, which emphasize the instrumental aspects of caring and care tasks; (b) personal and responsive relationships, which emphasize understanding the residents as a person with particular needs, that are developed through conversations with residents and family; and (c) reciprocal relationships, which emphasize the role and needs of all residents, staff, and family members in creating a sense of community within the home, featuring negotiation and compromise within a context of trust. Although relationships and meaningful experiences for both residents and care providers vary across care settings and changing faces, personal relationships that are responsive and reciprocal deliver the most positive experiences for the residents (Brown-Wilson, 2008).

Koren (2010) and Bowers, Esmond, and Jacobson (2000) have identified two barriers to building meaningful relationships in care homes: organizational barriers, such as workload, time restraints, and staff turnover or inadequate staffing. Similarly, Abbot et al., (2017) concluded that residents’ preferences were likely to change based upon the quality of interaction between the resident and staff and the resident’s level of interest. Similar results were obtained in this study, where residents could not establish a bonding relationship with the nurses because of certain barriers such as lack of continuity of care or lack of time nurses had to spend with them.

Older residents who had a close intimate friend, that is, a confidant, maintained higher morale in the face of stresses than residents who lacked such a relationship. In contrast, residents without a confidant who decreased their interaction with nurses were depressed. These results support the view that people need some social attachments to be happy and they need these interactions to occur in a framework of long term, stable caring and concern. Having two as opposed to no close relationships may make a world of difference to the person’s health and happiness. Close relations with staff apparently substituted for relationships with offspring, at least in terms of preventing any significant loss of happiness.

The findings point to situations where residents actively seek opportunities to interact with staff in a more personal manner. Activities such as sitting in communal areas in the nursing home, sitting in the dining area during mealtimes, and going for a walk with a nurse maximize the interaction of the residents with staff, which has been observed in previous studies (Cook & Brown-Wilson, 2010; Davies & Nolan, 2004; Merla et al., 2018). Staff attitudes towards their interactions with residents are equally important. Nonverbal exchanges such as smiling or sharing personal stories can do much to offset some of the less positive aspects of living in a care home. They provide a framework for social conversations, which can in turn become a foundation for the development of personal relationships (Brown-Wilson & Davies, 2009; van Stenis et al., 2017). The theoretical framework selected by McGilton et al. (2003) indicated that if a care provider is reliable and empathic with a resident, and provides continuity of care, and if a supportive environment is available, relational care can be improved.

## Limitations

The findings of this study reported the experiences of cognitively intact residents who were generally positive people, had positive feelings about their experiences in the nursing home, and were successful at developing meaningful relationships with their care providers. Further research conducted with residents who have been less successful socially integrating in the nursing home may be helpful in generalizing the findings.

Moreover, the findings represent the perception of the older residents only. While the resident perception of relationship development is informative, conducting future research exploring dyadic understandings of relationship development may also be important.

## Conclusion

The ways residents define and develop relationships with staff is considerably complex. The findings presented in this study indicate that if nurses are truly to provide care with compassion and dignity, sustaining interpersonal relationships is essential. These relationships represented social ties that reinforced acceptance and contributed to friendship, belonging and reassurance. Nurses and other practitioners need additional guidance about interacting with older people in ways that best support relationships. Assisting the nurses to learn how to create close relationships with residents may be one way to enhance the possibility of relational practice/meaningful relationships developing, always contingent upon the residents’ wishes.

## Relevance to clinical practice

The findings reached in this study indicate that there needs to be recognition by the nurses that relationships and involvement with others is integral for nursing home quality of life. Nurses should incorporate active listening in nursing care and maintain reciprocal relationships with residents in order to improve their quality of life. All care staff should broaden the scope of nursing home activities and should customize to personal interests, needs, and life history relating to each older resident. Another important consideration is that perception of the residents as not only patients but also as persons who deserve to live the last stage of their life in dignity and to be treated with respect away from undermining conduct.
